# Effect of Genome and Environment on Metabolic and Inflammatory Profiles

**DOI:** 10.1371/journal.pone.0120898

**Published:** 2015-04-08

**Authors:** Marina Sirota, Gonneke Willemsen, Purnima Sundar, Steven J. Pitts, Shobha Potluri, Edi Prifti, Sean Kennedy, S. Dusko Ehrlich, Jacoline Neuteboom, Cornelis Kluft, Karen E. Malone, David R. Cox, Eco J. C. de Geus, Dorret I. Boomsma

**Affiliations:** 1 Rinat-Pfizer, South San Francisco, California, United States of America; 2 Department of Biological Psychology, VU University Amsterdam, Amsterdam, Netherlands; 3 INRA, Institut National de la Recherche Agronomique, Metagenopolis, Jouy-en-Josas, France; 4 Good Biomarker Sciences, Leiden, Netherlands; Centers for Disease Control and Prevention, UNITED STATES

## Abstract

Twin and family studies have established the contribution of genetic factors to variation in metabolic, hematologic and immunological parameters. The majority of these studies analyzed single or combined traits into pre-defined syndromes. In the present study, we explore an alternative multivariate approach in which a broad range of metabolic, hematologic, and immunological traits are analyzed simultaneously to determine the resemblance of monozygotic (MZ) twin pairs, twin-spouse pairs and unrelated, non-cohabiting individuals. A total of 517 participants from the Netherlands Twin Register, including 210 MZ twin pairs and 64 twin-spouse pairs, took part in the study. Data were collected on body composition, blood pressure, heart rate, and multiple biomarkers assessed in fasting blood samples, including lipid levels, glucose, insulin, liver enzymes, hematological measurements and cytokine levels. For all 51 measured traits, pair-wise Pearson correlations, correcting for family relatedness, were calculated across all the individuals in the cohort. Hierarchical clustering techniques were applied to group the measured traits into sub-clusters based on similarity. Sub-clusters were observed among metabolic traits and among inflammatory markers. We defined a phenotypic profile as the collection of all the traits measured for a given individual. Average within-pair similarity of phenotypic profiles was determined for the groups of MZ twin pairs, spouse pairs and pairs of unrelated individuals. The average similarity across the full phenotypic profile was higher for MZ twin pairs than for spouse pairs, and lowest for pairs of unrelated individuals. Cohabiting MZ twins were more similar in their phenotypic profile compared to MZ twins who no longer lived together. The correspondence in the phenotypic profile is therefore determined to a large degree by familial, mostly genetic, factors, while household factors contribute to a lesser degree to profile similarity.

## INTRODUCTION

Twin and family studies have been historically used to examine the effects of genetics and environment on a wide range of complex human phenotypic traits [1–4]. There has been an abundance of studies examining the influence of genetic factors on a large spectrum of cardiovascular, immunological and metabolic traits. For most physiological parameters significant heritability is demonstrated. For instance, heritability of blood pressure ranges from 30 to 60% [5,6], while the heritability estimate for BMI can be even higher, above 70% [7–9], though it may differ as a function of age and social situation [10]. Previous studies have also shown genetic influences on indices of pro-inflammatory state, with heritability estimates ranging between 20 and 45% [11,12]. These measurements, however, are not independent from one-another. For instance, the metabolic syndrome has been characterized as consisting of several well established risk factors for cardiovascular disease, whose co-variation is largely due to genetic factors [13]. A syndrome score reflecting all risk factors is often used, which can result in a loss of information. Moreover, the metabolic syndrome (obesity, hyperlipidemia, hyperglycemia and hypertension) is associated with many other traits that may also explain cardiovascular disease, but are not formally part of the syndrome. In general, many studies that assess a large range of correlated biomarkers tend to analyze them in a one-by-one fashion, which fails to take into account their often-substantial inter-dependence.

Over the last decade, increasing attention is given to the methodology for the joint analyses of multiple traits, including principal components, factor analyses and other structural equation modeling approaches and recently the application of item response theory [14–17]. However, some of these techniques are difficult to implement in studies of familial resemblance, when for example the number of traits is large relative to the number of families. Here, we propose an approach, in which we define a phenotypic profile of an individual as the collection of **all** quantitatively measured traits for that individual and apply correlation metrics to capture the pairwise similarity across a large number of quantitative traits. By comparing the pairwise similarity of the phenotypic profiles within specific groups, which share genes and environment to a different extent, the underlying cause of variation in the phenotypic profile can be explored. To illustrate this, we applied our approach (see Methods) to a range of metabolic, hematological, and immunological traits (Table 1), which were collected in a large cohort of monozygotic (MZ) twins and their spouses from the Netherlands Twin Register. We explored positive and negative linear relationships between the traits correcting for age, sex and the family structure (see Methods) and used a hierarchical clustering approach to further test and visualize the relationships. These relationships can be used in the future to inform study design and account for the necessary covariates. They also provide insight into which traits are most informative to measure.

**Table 1:** List of measured traits with their median, mean and standard deviation.

**Variable**	**Description**	**Median**	**Mean**	**Standard Deviation**
Age	Age	34.0	33.88	11.58
ALT	Alanine transaminase (U/L)	18.0	19.14	7.48
AST	Aspartate aminotransferase (U/L)	23.0	24.90	8.30
basophils	Basophils (10*9/L)	0.0	0.02	0.01
BMI	Body mass index (kg/m2)	23.6	24.25	3.79
cholesterol	Total cholesterol (mmol/L)	4.8	4.90	0.91
cotinine	Cotinine	0.0	83.51	254.53
creatinine	Creatinine (mol/L)	80.0	80.13	12.17
CRP	C-reactive protein (mg/L)	1.2	2.25	2.81
DBP	Diastolic blood pressure (mmHg)	80.0	80.39	10.79
eosinophils	Eosinophils (10*9/L)	0.2	0.19	0.13
fibrinogen	Fibrinogen (g/L)	3.1	3.20	0.63
GGT	Gamma glutamyl transpeptidase (U/L)	19.0	23.96	15.98
glucose	Glucose (mmol/L)	4.7	4.76	0.47
GMCSF	Granulocyte-macrophage colony-stimulating factor (pg/ml)	0.0	0.34	0.82
HCT	Hematocrit (ratio)	0.4	0.42	0.03
HDL	HDL cholesterol (mmol/L)	1.4	1.44	0.37
height	Height (cm)	173.0	173.40	9.37
HGB	Hemoglobin (mmol/L)	8.7	8.80	0.77
hip	Hip circumference (cm)	102.0	102.72	7.74
HR	Heart rate (bpm)	71.0	71.82	11.81
IFNg	Interferon gamma (pg/ml)	1.9	2.37	2.22
IL10	Interleukin-10 (pg/ml)	1.7	4.53	10.89
IL12p70	Interleukin-12p70 (pg/ml)	2.3	10.14	38.60
IL1b	Interleukin-1 beta (pg/ml)	0.0	0.44	0.74
IL2	Interleukin-2 (pg/ml)	0.0	0.22	0.54
IL6	Interleukin-6 (pg/ml)	0.8	0.97	1.34
IL6R	Interleukin-6 receptor	380.5	392.57	117.44
IL8	Interleukin-8 (pg/ml)	2.7	2.64	1.21
insulin	Insulin (microIU/ml)	5.0	6.10	5.32
LDL	LDL cholesterol (mmol/L)	2.8	2.85	0.82
lymphocytes	Lymphocytes (10*9/L)	2.2	2.25	0.69
MCH	Mean corpuscular hemoglobin (fmol)	1.8	1.84	0.10
MCHC	Mean corpuscular hemoglobin concentration (mmol/L)	20.8	20.82	0.50
MCV	Mean corpuscular volume (fl)	88.3	88.32	4.15
monocytes	Monocytes (10*9/L)	0.5	0.55	0.17
MPV	Mean platelet volume (fl)	11.1	11.12	0.91
neutrophils	Neutrophils (10*9/L)	3.0	3.19	1.13
PCT	Platelet Count	0.3	0.28	0.06
PDW	Platelet distribution width	13.3	13.59	2.06
PLT	Platelets (10*9/L)	244.0	251.53	55.72
RBC	Red blood cell count (10*12/L)	4.8	4.79	0.42
RDWCV	Red cell distribution width, coefficient variation	12.9	13.00	0.83
RDWSD	Red cell distribution width, standard variation	40.9	41.06	2.38
SBP	Systolic blood pressure (mmHg)	127.0	128.87	15.15
telomerelength	Telomere length	2.6	2.66	0.40
TNFA	Tumor necrosis factor alpha (pg/ml)	6.3	6.66	1.96
triglycerides	Triglycerides (mmol/L)	1.1	1.34	0.78
waist	Waist circumference (cm)	78.0	80.02	11.49
WBC	White blood cell count (10*9/L)	6.0	6.18	1.54
weight	Weight (kg)	71.4	73.17	14.42
WHR	Waist to hip ratio (cm/cm)	0.8	0.78	0.08

**Fig 1:**
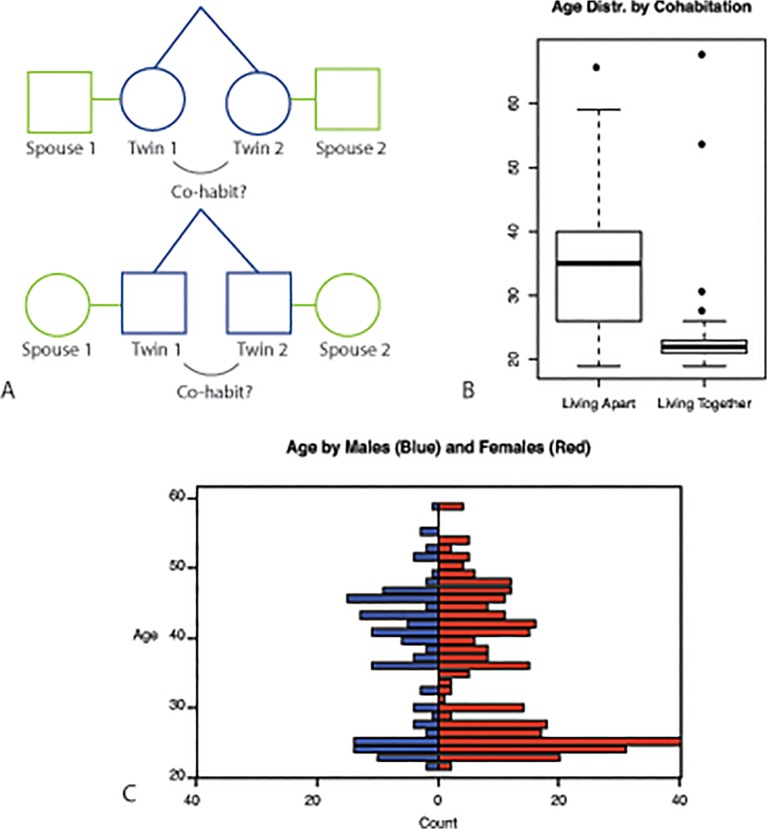
Study overview and participant characterization. A) Study Design. Pairs of twins were recruited for this study. A proportion of the cohort consists of twins who are co-habiting. Spouses were also recruited to index resemblance through shared household factors; B) Age distribution in twin study participants stratified by co-habitation status; C) Age distribution in study participants stratified by sex.

In addition to studying relationships between the traits, we examine the similarity of specific phenotypic traits as well as phenotypic profiles across individuals. The inclusion of spouses, who share a household, in addition to MZ twin pairs, who share their genome, allow for an exploration of genetic and environmental influences on the phenotypic profile by determining the similarity in profile for MZ pairs, twin-spouse pairs and unrelated individuals (who share neither genes nor environment). Family members may resemble one-another due to shared genes and shared environments. In the classical twin design the similarity in MZ and DZ twins is compared to disentangle the effect of these genetic and the shared environmental influences. Here, we use an alternative method by contrasting MZ twin similarity to the similarity in spouse pairs, who share a household but can be considered not to share a genetic background [18]. A higher profile similarity in MZ twins compared to unrelated individuals indicates familial, genetic and/or shared—childhood- environment influences (Fig. 1A). A staircase pattern with similarity being highest in MZ twins, lower in spouses, and much lower among unrelated individuals, would indicate influence of shared environment, where spouse resemblance provides an upper limit for household effects if phenotypic assortative mating is also present. In contrast, a pattern in which the similarity for spouses is comparable to unrelated individuals, with MZ twin similarity being higher, would indicate that household effects do not play a role. The importance of sharing a household is further explored by determining profile similarity in MZ twins who cohabit and in MZ twins who live separate from each other. If non-cohabiting twins are less alike in their profile than cohabiting twins, this points to an influence of shared household factors, although we recognize that cohabiting twins tend to be younger.

## MATERIALS AND METHODS

### Participants

The participants in this study were recruited from the Adult Netherlands Twin Register (ANTR), a large twin-family based register for the study of individual differences in health, lifestyle and personality across the lifespan [19,20]. Adult participants were invited in the present study when they did not report any diseases related to the immune system, endocrine system or gastrointestinal tract in earlier survey studies. The study mainly targeted MZ twin pairs and their spouses, but in the initial pilot phase of the study other healthy family members were also invited. Invitation letters to NTR participants were followed by a phone call. Of those participants reached by phone, 71% agreed to take part in the study. The final cohort consisted of 517 individuals (67% female): 447 twins (210 pairs and 27 unpaired twins), 64 spouses, 1 sibling, 2 fathers, 2 mothers and 1 singleton. The age of the participants ranges from 19 to 68 (mean 33.9, standard deviation 11.9). The age bimodal distribution stratified on sex (more females than males for the younger individuals) and cohabitation status are shown in Fig. 1C and 1B. All data are used for the analysis of relationships among traits. The heritability analysis focuses on the 210 MZ twin pairs, and 64 twin-spouse pairs included within this cohort. Within the 210 pairs of twins, 57 were cohabiting at the time of the sampling and 153 pairs were not. The zygosity was confirmed by DNA markers. Ethical approval was obtained from the Central Ethics Committee on Research Involving Human Subjects of the VU University Medical Center, Amsterdam. Written informed consent was obtained from all participants. The study was approved by the Central Ethics Committee on Research Involving Human Subjects of the VU University Medical Centre, Amsterdam, an Institutional Review Board certified by the U.S. Office of Human Research Protections (IRB number IRB-2991 under Federal-wide Assurance-3703; IRB/institute codes, NTR 03–180).

### Data collection

Participants were visited between 7 and 10 AM at home or, in a few cases, at work. They were instructed to be fasting and avoid exercise as of the night before and to refrain from smoking and, when possible, medication prior to the visit. Fertile women were bled on a fixed day (day 3) of their menstrual cycle, or during the placebo week of their birth control hormone regimen, if feasible. During the visit, participants were asked to provide information on fasting status, general health, medication use and lifestyle (such a smoking status, diet).

#### Body composition indices

Height, weight, waist and hip circumference were determined, which were used to calculate body mass index (BMI) and waist-to-hip ration (WHR).

#### Cardiovascular measurements

Using an OMRON HEM907 blood pressure device, systolic blood pressure (SBP), diastolic blood pressure (DBP) and heart rate (HR) were measured twice, before and after the blood sampling. In the present study we used the average SBP, DBP and HR across the two measurements.

#### Blood Sample Collection

Using the Vacutainer system, 8 blood tubes were collected: 2 x 9 ml EDTA, 1 x 9 ml Li heparine, 1 x 9 ml Na heparine, 1 x 4.5 ml CTAD, 1 x 2.5 ml PAX, 1 x 5 ml serum and 1 x 2 ml EDTA. To prevent clotting, all tubes were inverted gently 8–10 times immediately after collection [21].

#### Lipids

Total cholesterol, HDL-cholesterol and triglycerides were determined in plasma from a lithium heparin tube, which was stored in melting ice during transport and processed at the laboratory within 6 hours of transport. After centrifugation of the tube for 15 minutes at 1000x g at 4° C, heparin plasma was obtained and divided into 8 subsamples of 0.5 ml, snap-frozen and stored at—30° C. One these subsamples provided lipid levels, using Vitros 250 total cholesterol, Vitros 250 direct HDL cholesterol and Vitros 250 Triglycerides assays (Johnson & Johnson, Rochester, USA) respectively. LDL-cholesterol was calculated using the Friedewald Equation: LDL = Total cholesterol—HDL—(Triglycerides/5) [22].

#### Glucose metabolism

Insulin was measured using the Immulite 1000 Insulin Method (Diagnostic Product Corporation, Los Angeles, USA) and glucose was measured using the Vitros 250 Glucose assay (Johnson & Johnson, Rochester, USA).

*Liver enzymes*. GGT, ALT and AST levels in units per liter (U/L) were determined for one of the lithium heparin plasma aliquots, using Vitros assays (Vitros 250, Ortho-Phenotypic Diagnostics; Johnson & Johnson, Rochester). Please see Table 1 for a complete list.

#### Hematology

The 2 ml EDTA tube was stored at room temperature during transport and used to obtain a hematological profile via the Coulter system (Coulter Corporation, Miami, USA). Parameters provided were white blood cell count (WBC), numbers of neutrophils, lymphocytes, monocytes, eosinophils, basophils as well as red blood cell count, hemoglobulin, hematocrit, mean corpuscular volume (MCV), mean corpuscular hemoglobin (MCH), mean corpuscular hemoglobin concentration (MCHC), red cell distribution width (RDW), platelet count (PCT) and mean platelet volume (MPV). Please see Table 1 for a complete list.

#### Cytokines

Nine cytokines were determined in blood from one of the EDTA tubes, which were stored in melting ice during transport. Upon arrival at the laboratory, the EDTA tubes were centrifuged for 20 minutes at 2000x g at 4° C. EDTA plasma was harvested and aliquoted (0.5 ml), snapfrozen in dry ice, and stored at—30° C. Using the Meso Scale Discovery platform and one of the EDTA plasma aliquots, the levels of IL6, TNF-α, GMCSF, IFN-γ, IL10, IL12p70, IL1b, IL2, and IL8 were determined. Please see Table 1 for a complete list.

#### C-reactive protein (CRP)

CRP was obtained from one of the lithium heparin aliquots using the Immulite 1000 CRP assay (Diagnostic Product Corporation, USA).

*Fibrinogen*. Fibrinogen levels were obtained from CTAD plasma. The 4.5 ml CTAD tube was stored during transport in melting ice and upon arrival at the laboratory, centrifuged for 20 minutes at 2000x g at 4° C, after which citrated plasma was harvested from the buffy coat and red blood cells, aliquoted (0.5 ml), snap frozen in dry ice, and stored at—30° C. One of these aliquots was used to obtain fibrinogen levels on a STA Compact Analyzer (Diagnostica Stago, France), using STA Fibrinogen (Diagnostica Stago, France).

#### Data Analysis

Data were inspected and extreme outliers (defined below) were removed. For individuals who were taking antihypertensive medication (N = 24), systolic blood pressure was increased by 14 mmHg and diastolic blood pressure by 10 mmHg [23]. Triglyceride levels were set to missing when non-fasting (N = 8), and 0.74 mmol/l was added to LDL cholesterol and total cholesterol when LDL-lowering medication (statins, N = 8) were used [24]. Glucose and insulin levels were set to missing when non-fasting (N = 8) and additionally when on diabetes medication (N = 4). In the case of glucocorticoid or anti-inflammatory medication, cytokine levels were set to missing (N = 8).

Based on inspection of the distribution of the hematology data we set data to missing when white blood cell count was above 12 (N = 7), neutrophil count above 8 (N = 5), lymphocyte count above 5 (N = 2), monocyte count above 1.1 (N = 6) and basophil count above 10 (N = 1). We further set to missing MCHC when above 24 (N = 1), HCT above. 6 (N = 1), HGB above 14 (N = 1) and red blood cell count above 7 (N = 1). Values were set to missing when individuals were using anti-inflammatory or endocrine medication or had values above 5 for CRP (N = 21), IL6 (N = 9), TNFalpha (N = 22), IFNgamma (N = 17), or above 6 for fibrinogen (N = 7), above 110 for IL10 (N = 24), above 500 for IL12p70 (N = 16), and above 5 for IL1b (N = 11), IL2 (N = 10), and IL8 (N = 14).

Table 1 provides an overview of the 51 quantitatively measured traits included in the analyses. It’s important to note that several cytokines have skewed distributions. The phenomenon of such tailed distributions might be due to transient or chronic immunological or inflammatory processes. Many of the observed values are below detection limit (29% for Il-6; 77% for GMCSF, 19% for IFN-gamma, 65% for Il-1-beta and 83% for Il-2) resulting in a difference between means and median measurements, which are also reported in Table 1. We recognize that this is a limitation of the measurement modalities that are applied here In order to correct for age and sex variability in the cohort, we computed linear regression residuals for each trait. The distributions of these residual values per trait are shown in S1 Fig.

First, we tested the correlation between all traits in the full sample of 517 individuals by computing partial pairwise Pearson correlations across all the individuals in the cohort (ppcor Package in R), in which we controlled for the relatedness between individuals by taking into account individuals from the same family and their relationship to each other (twin or spouse). In total, 1275 pairwise trait correlations were generated. Based on Bonferroni correction for multiple hypothesis testing, the threshold for significance was < 3.92e-05. We applied a hierarchical clustering technique using the complete linkage method [25] to group the measured traits into sub-clusters based on 1—correlation as a distance metric. Complete linkage method is a type of agglomerative clustering where each phenotypic trait starts in its own cluster, and consequently pairs of clusters are merged as one moves up the hierarchy based on the 1—partial Pearson correlation value as a distance metric.

To establish the relative contribution of genetic and household factors to the variance in all individual traits, we computed the pair-wise correlation between MZ twin pairs (living together and living apart), twin-spouse pairs as well as randomly sampled unrelated individuals as a measure of heritability for each trait. The unrelated individual pairs were constructed by randomly sampling from the cohort for each person excluding their twin and spouse from the cohort. For each of the 51 measured traits we computed: **twin_correlation_cohab** = correlation(t[twin1], t[twin2]) where twin1 and twin 2 are cohabiting, **twin_correlation_not_cohab** = correlation(t[twin1], t[twin2]), where twin1 and twin2 are not cohabiting, **twin_spouse_correlation** = correlation(t[twin], t[spouse]) and **unrelated_individuals** = correlation(t[indiv1], t[indiv2]) where indiv1 and indiv2 are chosen at random.

The phenotypic profile p of an individual was defined as a vector of all the phenotypic measurements (n = 51) for that individual. Pearson correlation, capturing the resemblance between pairs of vectors, is used a measure of similarity of two phenotypic profiles. Pearson correlation coefficient is a measure of the linear correlation between two variables X and Y, giving a value between +1 and −1 inclusive, where 1 is total positive correlation, 0 is no correlation, and −1 is total negative correlation. It is furthermore defined as the covariance of the two variables divided by the product of their standard deviations. Pairwise profile similarity between all pairs of individuals was determined for four subgroups: MZ twin pairs sharing (57) or not-sharing a household (153), twin-spouse pairs (64) and pairs of unrelated individuals (132,596). Here for each within pair comparison in our four groups we compute a similarity score s = correlation(p_indiv1, p_indiv2).

All statistical analyses were performed in R.

## RESULTS

### Characterization and Clustering of the Phenotypic Traits

Table 1 includes all the measured traits used for the analysis with their median, mean and standard deviation in the dataset. Using the data from the 517 individuals in our sample, we computed partial Pearson correlations, correcting for the effect of family relatedness, for the 51 measured traits. Out of the 1,275 relationships tested, 169 were significant at p < 0.05/1275~4e05 (correlations ranged between -0.558 to +0.996, see S1 Table).

**Fig 2:**
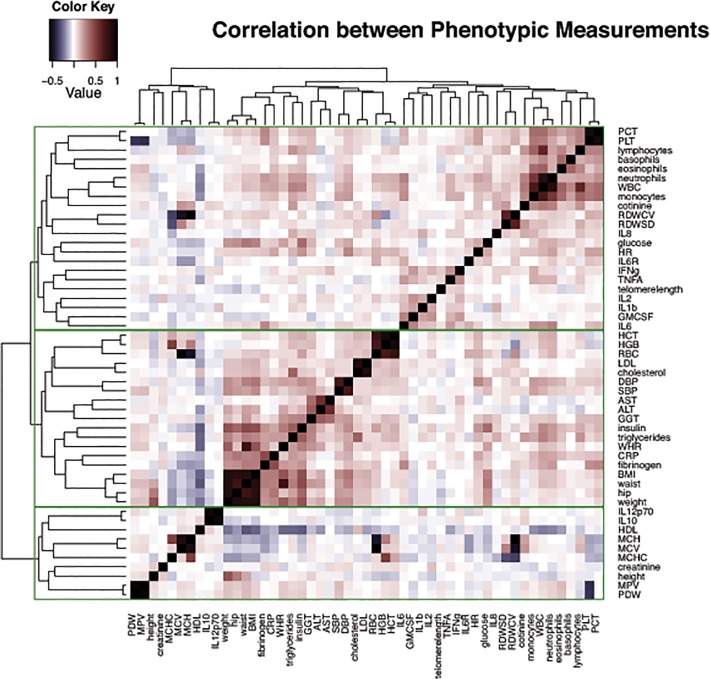
Hierarchical clustering of phenotypic measurements. This figure shows the clustering of the phenotypic measurements based on their correlations with each other. Correlations between all pairs of phenotypes are computed, while partialling out the effect related to family membership. Positive correlations are shown in red, negative correlations in purple. The clusters are shown in green.

We applied hierarchical clustering to elucidate and visualize relationships between the measured traits (Fig. 2). Many of the body composition measurements such as weight, waist, hip, waist to hip ratio were strongly correlated with blood pressure, cholesterol levels, insulin and glucose measurements and inversely correlated with HDL levels, defining the metabolic syndrome. As to be expected, hematocrit and hemoglobin measurements were highly correlated (correlation coefficient 0.89–0.93, p < 4e05) with other types of red blood cell count measurements and inversely related to measures of red blood cell volume and size. Inflammatory markers such as white blood cell count (WBC), neutrophil, lymphocyte, eosinophil, monocyte and basophil counts were correlated with IL6 cytokine levels (correlation coefficients 0.24–0.28, p < 4e05). Those traits also correlated positively with platelet counts (PCT, PLT, correlation coefficient 0.27–0.35, p < 4e05). A positive correlation was observed between IFN-γ and TNF-α (correlation coefficient = 0.25, p < 4e05), which are known to act synergistically during inflammation[26]. IFN-γ and TNF-α also cluster together with IL2, IL1b and GMCSF, which are known to have common mechanisms in response to co-stimulatory signals in T-cells [27]. Levels of IL10 and IL12, two cytokines which are both involved in Th1 T-cell differentiation [28], were highly correlated (correlation coefficient = 0.95, p < 4E-05).

Interestingly, we observed a weak, but significant positive correlation between cotinin, an alkaloid found in tobacco and a metabolite of nicotine, and various inflammatory markers (correlation coefficient 0.23–0.32, p < 4e05). We also observed a weak positive correlation between heart rate and glucose as well as insulin levels (correlation coefficient = 0.19, p < 4e05). Other studies have demonstrated that an increased heart rate at rest is associated with glucose levels [29,30].

A strong correlation was seen between fibrinogen, a coagulation marker, and CRP, a general measure of inflammation (correlation coefficient = 0.46, p < 4e05). The liver synthesizes both CRP and fibrinogen and in this study they were both positively correlated with body composition (weight, waist and BMI, correlation coefficient 0.24–0.3, p < 4e05). Fibrinogen was also positively correlated with other inflammatory markers (WBC and neutrophil counts, IL6 levels, correlation coefficients 0.19–0.26, p < 4e05).

Weak positive correlations were also seen for white blood cell count, an inflammatory marker, with insulin and triglyceride levels (correlation coefficients 0.27–0.29, p < 4E-05) and negatively correlated with HDL (correlation coefficient -0.22, p < 4e05).

### Trait similarity across twins, spouses and unrelated individuals

**Fig 3:**
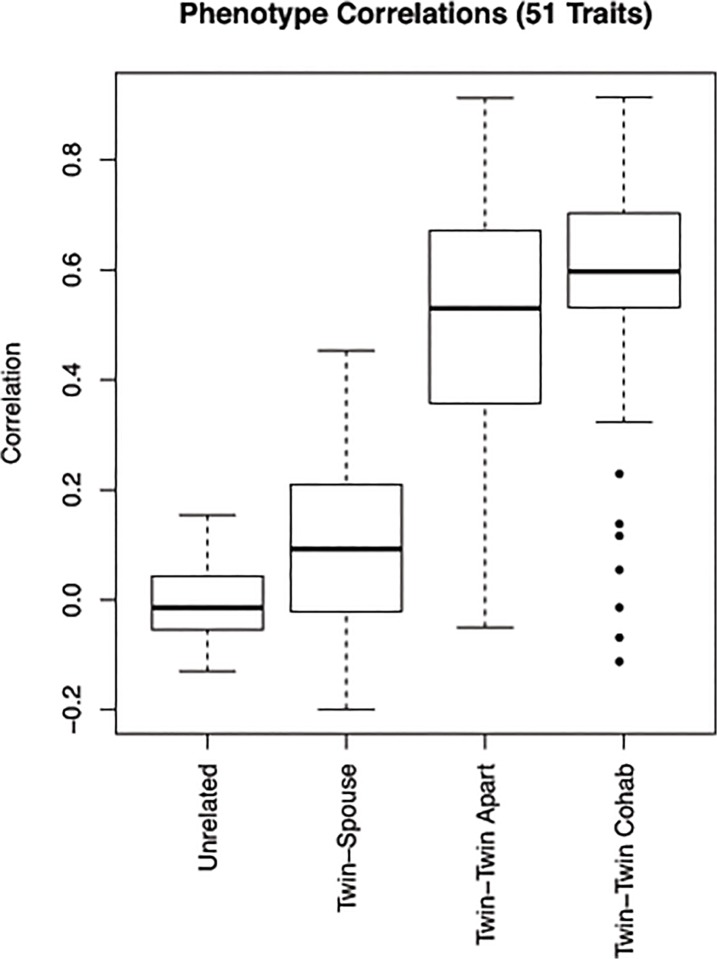
Trait similarity across twins, spouses and unrelated individuals. This figure shows the boxplot with distribution across the 51 measured traits showing correlation in twins (cohabiting and not co-habiting), twin-spouse pairs and unrelated individuals. Average within pair correlation for the MZ twins living together is 0.56 (living together), for MZ twins living apart 0.48 (living apart), for twin-spouse pairs 0.08 and for non-related individuals 0.

In order to establish the relative importance of genetic and household factors for each trait, we examined the pairwise similarity for each of the separate traits in four groups—unrelated individuals, spouse pairs, non-cohabiting MZ twins and cohabiting MZ twins respectively. Fig. 3 shows the distribution of the correlation coefficients for the 51 traits in MZ twin pairs living together (mean = 0.56), MZ twin pairs living apart (mean = 0.48), spouse pairs (mean = 0.08) and pairs of unrelated individuals (mean = 0.00). Although we do see a higher average correlation across the traits in MZ twins who are living together than for those living apart, the difference is not statistically significant. The traits are much more correlated in both twin groups than in twin-spouse pairs or unrelated individuals (all comparisons were highly significant, p < 2.2e16). In comparison to unrelated individuals (mean = 0.00) the distribution of correlations between twin-spouse (mean = 0.08) was significantly higher (p-value = 0.0001) demonstrating the presence of a small environmental effect.

**Fig 4:**
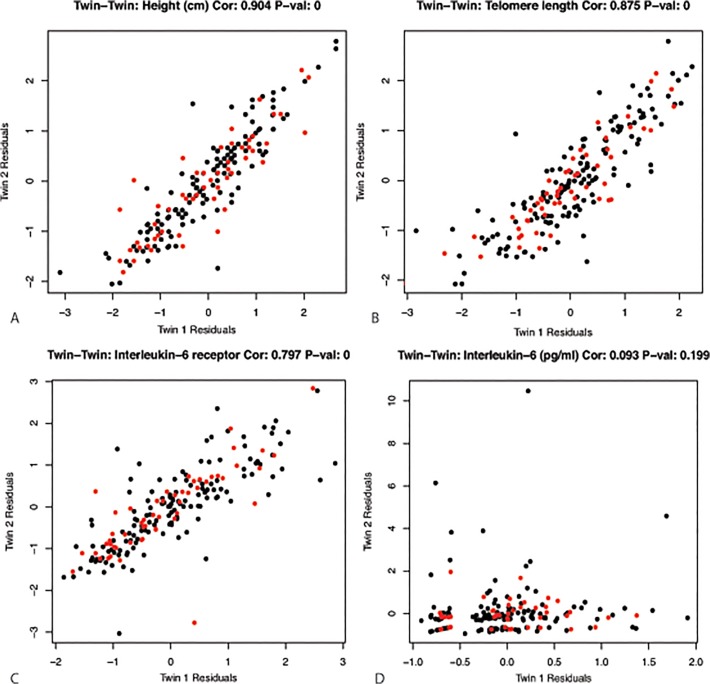
Examples of traits and their concordance in twins. MZ twin pair concordance for A) height, B) telomere length, C) IL6 receptor, D) IL6. In black the correlations for twins living apart, in red the correlations for twins living together. Values shown are sex and age corrected residuals (see Methods).

MZ pair resemblance for certain traits is more striking than for others (Fig. 4A, Table 2). As expected [31], highest resemblance was found for height (correlation coefficient = 0.9, p-value < 2.2e16). In addition, telomere length, which has been previously associated with normal ageing[32,33], was highly correlated (correlation coefficient = 0.875, p-value < 2.2e16) in MZ twins (Fig. 4B). This is in line with a previous study by Broer et al. [34], who also demonstrated high heritability for telomere length. We further observed a high MZ twin correlation for the plasma level of the soluble IL6 receptor (correlation coefficient = 0.8, p-value < 2.2e16), whereas the resemblance for plasma levels of the cytokine itself was very low (correlation coefficient = 0.09, p-value = ns) Fig. 4C and 4D.

**Table 2:** Pair-wise correlations (confidence intervals are provided in S2 Table) for each trait in each of the four comparison groups (MZ twin pairs living together, MZ twin pairs living apart, twin-spouse pairs, unrelated individuals) ordered by concordance in twins living together.

		Twin-Twin Living Together		Twin-Twn Living Apart		Twin-Spouse		Unrelated	
**Variable**	Description	Correlation	P-Value	Correlation	P-Value	Correlation	P-Value	Correlation	P-Value
telomerelength	Telomere length	0.9133	0.0000*	0.8561	0.0000*	-0.0279	0.8270	0.0003	0.9963
cotinine	Cotinine	0.9001	0.0000*	0.3469	0.0000*	0.2950	0.0189	0.0739	0.2897
height	Height (cm)	0.8841	0.0000*	0.9126	0.0000*	0.1451	0.2526	0.0440	0.5265
triglycerides	Triglycerides (mmol/L)	0.8838	0.0000*	0.3777	0.0000*	0.0783	0.5454	-0.0491	0.4885
hip	Hip circumference (cm)	0.8035	0.0000*	0.6716	0.0000*	0.3027	0.0159	-0.0613	0.3812
BMI	Body mass index (kg/m2)	0.8007	0.0000*	0.6239	0.0000*	0.2560	0.0464	-0.0252	0.7193
weight	Weight (kg)	0.7901	0.0000*	0.6832	0.0000*	0.2053	0.1125	-0.0968	0.1661
IL6R	Interleukin-6 receptor	0.7794	0.0000*	0.8038	0.0000*	0.2900	0.0259	-0.1104	0.1189
waist	Waist circumference (cm)	0.7671	0.0000*	0.5477	0.0000*	0.1174	0.3595	-0.0626	0.3705
PCT	Platelet Cout	0.7504	0.0000*	0.6870	0.0000*	0.1156	0.3628	-0.0487	0.4870
MCV	Mean corpuscular volume (fl)	0.7396	0.0000*	0.7561	0.0000*	0.0733	0.5646	0.1344	0.0542
basophils	Basophils (10*9/L)	0.7245	0.0000*	0.6079	0.0000*	0.0254	0.8423	0.0044	0.9500
LDL	LDL cholesterol (mmol/L)	0.7096	0.0000*	0.7039	0.0000*	0.0651	0.6093	-0.0026	0.9699
MPV	Mean platelet volume (fl)	0.6957	0.0000*	0.7703	0.0000*	-0.0880	0.4893	-0.0399	0.5691
RDWSD	Red cell distribution width, standard variation	0.6936	0.0000*	0.6657	0.0000*	0.0309	0.8086	0.0558	0.4257
PDW	Platelet distribution width	0.6918	0.0000*	0.7456	0.0000*	-0.0893	0.4828	-0.0138	0.8437
PLT	Platelets (10*9/L)	0.6847	0.0000*	0.6708	0.0000*	0.1011	0.4265	-0.0413	0.5556
cholesterol	Total cholesterol (mmol/L)	0.6684	0.0000*	0.6464	0.0000*	-0.0132	0.9176	0.0593	0.3961
MCH	Mean corpuscular hemoglobin (fmol)	0.6662	0.0000*	0.7019	0.0000*	0.0012	0.9925	-0.0252	0.7189
creatinine	Creatinine (mol/L)	0.6657	0.0000*	0.6441	0.0000*	0.3282	0.0081	0.0972	0.1635
HR	Heart rate (bpm)	0.6395	0.0000*	0.3131	0.0001*	0.0378	0.7686	0.0119	0.8643
DBP	Diastolic blood pressure (mmHg)	0.6323	0.0000*	0.5213	0.0000*	0.0518	0.6868	-0.0558	0.4214
eosinophils	Eosinophils (10*9/L)	0.6233	0.0000*	0.4949	0.0000*	0.0134	0.9163	-0.0014	0.9838
WHR	Waist to hip ratio (cm/cm)	0.6026	0.0000*	0.4095	0.0000*	-0.1052	0.4121	-0.0575	0.4118
WBC	White blood cell count (10*9/L)	0.6001	0.0000*	0.5301	0.0000*	0.2986	0.0165	-0.0797	0.2634
lymphocytes	Lymphocytes (10*9/L)	0.5975	0.0000*	0.5353	0.0000*	0.2148	0.0909	-0.0489	0.4875
SBP	Systolic blood pressure (mmHg)	0.5948	0.0000*	0.5810	0.0000*	0.2139	0.0923	-0.0775	0.2636
insulin	Insulin (microIU/ml)	0.5925	0.0000*	0.3254	0.0001*	0.1678	0.2041	-0.0608	0.3940
fibrinogen	Fibrinogen (g/L)	0.5886	0.0000*	0.4423	0.0000*	-0.0080	0.9522	-0.0248	0.7350
monocytes	Monocytes (10*9/L)	0.5873	0.0000*	0.6474	0.0000*	0.0928	0.4693	0.1068	0.1314
neutrophils	Neutrophils (10*9/L)	0.5800	0.0000*	0.3687	0.0000*	0.2512	0.0453	-0.0529	0.4556
CRP	C-reactive protein (mg/L)	0.5730	0.0000*	0.3200	0.0002*	-0.1983	0.1287	0.0936	0.2012
RDWCV	Red cell distribution width, coefficient variation	0.5708	0.0000*	0.5665	0.0000*	-0.0895	0.4821	0.0752	0.2826
HDL	HDL cholesterol (mmol/L)	0.5697	0.0000*	0.7372	0.0000*	0.0498	0.6960	-0.0147	0.8331
GGT	Gamma glutamyl transpeptidase (U/L)	0.5630	0.0000*	0.5487	0.0000*	-0.0277	0.8277	0.0488	0.4853
MCHC	Mean corpuscular hemoglobin concentration (mmol/L)	0.5510	0.0000*	0.5076	0.0000*	-0.0445	0.7289	0.0966	0.1670
RBC	Red blood cell count (10*12/L)	0.5446	0.0000*	0.6880	0.0000*	0.2382	0.0580	0.0722	0.3035
glucose	Glucose (mmol/L)	0.5342	0.0000*	0.4509	0.0000*	0.2283	0.0794	-0.1023	0.1496
HGB	Hemoglobin (mmol/L)	0.5285	0.0000*	0.4730	0.0000*	0.1138	0.3708	0.0395	0.5735
HCT	Hematocrit (ratio)	0.4834	0.0002*	0.5137	0.0000*	0.0950	0.4553	0.0799	0.2548
ALT	Alanine transaminase (U/L)	0.4445	0.0006*	0.3756	0.0000*	0.2899	0.0201	0.0149	0.8313
AST	Aspartate aminotransferase (U/L)	0.3495	0.0077	0.3411	0.0000*	0.1013	0.4297	0.0137	0.8452
IFNg	Interferon gamma (pg/ml)	0.3270	0.0205	0.2404	0.0051	0.1498	0.2704	-0.0675	0.3611
IL8	Interleukin-8 (pg/ml)	0.3237	0.0193	0.4329	0.0000*	0.4530	0.0005*	-0.0256	0.7236
GMCSF	Granulocyte-macrophage colony-stimulating factor (pg/ml)	0.2370	0.0940	-0.0499	0.5600	-0.0292	0.8294	0.1685	0.0202
IL2	Interleukin-2 (pg/ml)	0.1461	0.2967	0.0960	0.2662	-0.1095	0.4263	0.1014	0.1651
IL6	Interleukin-6 (pg/ml)	0.1244	0.3846	0.0931	0.2703	-0.0508	0.7024	-0.0115	0.8736
TNFA	Tumor necrosis factor alpha (pg/ml)	0.0628	0.6651	0.1573	0.0655	0.1206	0.3851	0.0296	0.6872
IL1b	Interleukin-1 beta (pg/ml)	-0.0058	0.9682	0.1149	0.1894	0.1727	0.2117	-0.1439	0.0540
IL10	Interleukin-10 (pg/ml)	-0.0603	0.6773	-0.0322	0.7129	-0.0331	0.8107	-0.0425	0.5672
IL12p70	Interleukin-12p70 (pg/ml)	-0.1040	0.4769	-0.0482	0.5888	-0.1040	0.4632	-0.0093	0.9018

Significant p-values (p < 0.001) are denoted with a *.

Evidence for a moderate influence of shared genetic background is seen for other inflammatory markers, platelet measurements, HDL and LDL cholesterol, as well as total cholesterol and creatinine levels. We observed that blood pressure was more correlated in the MZ twins (correlation coefficient > 0.5, p-value < 2.2e16) than in spouses (correlation coefficient = 0.21, p-value = ns) and unrelated individuals (correlation coefficient = -0.08, p-value = ns), indicating genetic influences. These findings are consistent with previous work by Xu et al who studied the genetics and environmental effects on BP variability [35]. MZ correlations in a similar range were observed for liver enzymes, heart rate and GGT, but correlations were lower for the other liver enzymes and cytokines (Table 2).

Interestingly, several variables were more correlated in twins living together than those living apart, signifying the role that the environment plays for those traits. The strongest such finding is for cotinine, a biomarker indicating tobacco exposure, for which the correlation coefficient is 0.9 (p-value < 2.2e16) in twins living together and 0.34 (p-value < 2.2e16) in twins living apart. Several other traits including triglyceride levels, waist circumference, heart rate and insulin levels showed a higher concordance in twins living together than those living apart and might have a larger environmental impact.

When examining how correlated these individual traits are in twin-spouse pairs and unrelated individuals (Table 2), only IL8 levels were significantly correlated in twin-spouse pairs (correlation coefficient = 0.45 and p = 0.0005). A few other traits were nominally significantly correlated in twin-spouse pairs including creatinine levels (correlation coefficient = 0.29, p-value = 0.02), hip circumference (correlation coefficient = 0.3, p-value = 0.02), white blood cell count (correlation coefficient = 0.3, p-value = 0.02) and cotinine levels (correlation coefficient = 0.3, p-value = 0.02).

### Phenotype similarity across twins, spouses and unrelated individuals

**Fig 5:**
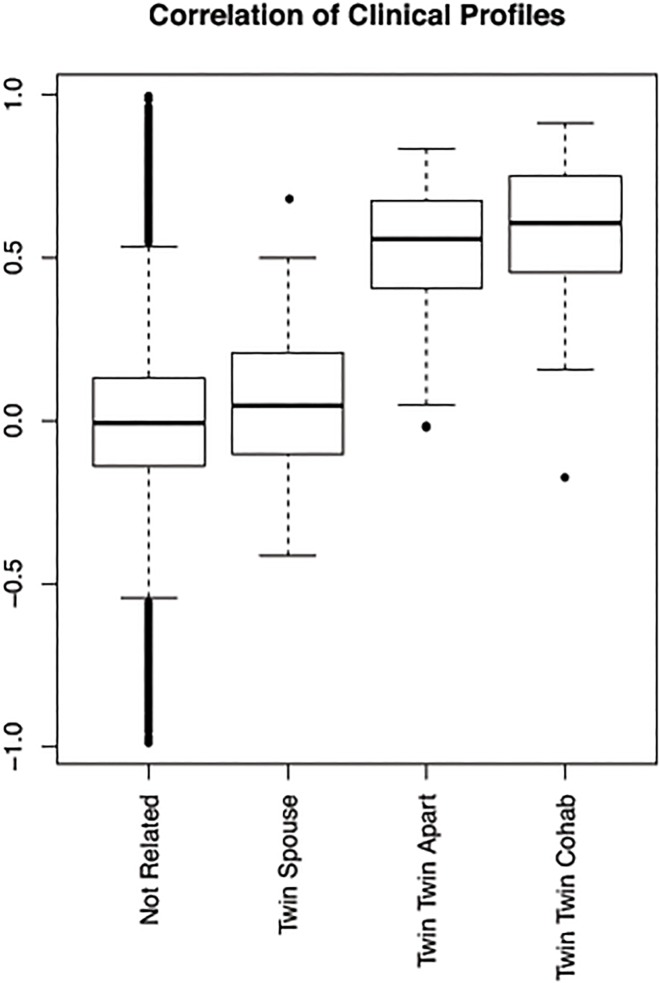
Correlation of phenotypic profiles across twins, spouses and unrelated individuals. This boxplot shows the pairwise correlations of phenotypic profiles between individuals: cohabiting MZ twins (57 pairs), MZ twins living apart (153 pairs), twin-spouse pairs (64 pairs) and non-related individuals (132596 pairs).

Fig. 5 summarizes the phenotypic profile similarity for unrelated individuals, spouse pairs, non-cohabiting MZ twins and cohabiting MZ twins respectively. The 57 cohabiting MZ pairs are younger and more often male than the MZ twin pairs who live separately (Fig. 1C, mean 23.3 vs. 35.4 years old, 63% vs. 73% female). However, all the measurements in the analysis have been corrected for sex and age.

We observe a significantly higher similarity in the profiles of twins than in twin-spouse pairs (average profile correlation = 0.54 versus 0.06, p-value < 2.2e16), which suggests a strong genetic influence on the observed phenotypic profiles. In order to validate this finding, we examined how closely twins would cluster together based on their phenotypic profiles and compared results on the actual data to those computed on a randomly permuted dataset (S2 Fig.). For nearly half of the twin pairs, the two members have most correlated profiles in the cohort relative to other individuals. In the majority of the pairs, starting with twin 1, twin 2 is in the top five most similar with regard to the phenotypic profile. Comparing the twin pairs who live together with the twin pairs who live apart, we observe a higher phenotypic profile correlation in the cohabiting group (average correlation 0.58 and 0.52 respectively, p-value = 0.01) suggesting an additional role for the environment in the phenotypic profile. The higher phenotypic profile correlations for the spouse pairs versus the unrelated pairs (average correlation = 0.06 versus -0.0023, p-value = 0.0007) provide further support for this small role for household factors in influencing one’s phenotypic profile.

## DISCUSSION

In this study we use correlation approaches to explore the similarity of genetically related individuals or individuals sharing a household across a large set of metabolic, hematological and immunological traits. In addition we identify known and novel relationships between the measured phenotypes. The unique design of the study, including twins as well as their spouses, allows us to study the genetic and household effects of the measured phenotypic traits. We define the concept of a phenotypic profile of an individual allowing us to look at multivariate relationships simultaneously to determine the resemblance of monozygotic twins in comparison to their spouses and unrelated individuals.

Looking at the overall relationships among all traits several interesting clusters could be identified. Many of the inflammatory markers were highly correlated across this healthy population based cohort. The overall white blood cell count as well as levels of various types of white blood cells (neutrophils, lymphocytes, monocytes) correlated with levels of IL6, a cytokine secreted by T-cells and macrophages to stimulate immune response. As expected, many of the metabolic phenotypes including weight, BMI, and waist circumference, were also correlated with each other and other traits such as LDL, total cholesterol, glucose and insulin levels.

Some traits such as heart rate, liver enzyme levels, fibrinogen and CRP were correlated with both metabolic (BMI, waist, weight, triglyceride and glucose levels) and inflammatory markers (IL6, WBC, neutrophils), which have also been seen by Il'yasova et al in 2008 [36]. The biological links between metabolism and inflammation, mostly through lipid-activated nuclear receptors (LXR) and peroxisome proliferator-activated receptors (PPARs) have been explored extensively in previous studies [37,38]. The relationship that we observe between fibrinogen and CRP has also been previously noted[39]. There are several novel relationships that are observed such as cotinine and inflammation (WBC, neutrophils, lymphocytes, monocytes). This study thus captures associations between metabolism and inflammation through phenotypic measurements of healthy individuals. This type of analysis can also be extended to individuals with disease to see how the network of phenotypic measurements and the relationships between them differ as a function of disease status or by therapeutic interventions.

The unique design of this study allows us to determine the relative contribution of genetic and household factors to familial resemblance in the metabolic, hematological and immunological traits. In general, when comparing the intra-pair resemblances of the traits of cohabiting MZ twins, resemblance can be a function of both their genes and shared environment, whereas the resemblance of non-cohabiting MZ twins provides an upper limit of the heritability of the trait. In twin-spouse pairs, who share environment only, the resemblance is caused by household factors. Finally, we compare twin-twin and twin-spouse correlations to those obtained in unrelated individuals who share neither genes nor environment.

Results of the comparison of similarity across MZ twin pairs, spouse pairs and pairs of unrelated individuals clearly demonstrated the importance of genetic factors on phenotypic profiles as well as many of the individual phenotypic traits in healthy individuals. The profiles among MZ twins, either cohabiting or non-cohabiting, were significantly higher correlated than those of twin-spouse pairs or unrelated individuals, pointing to the importance of genetic factors although a lingering role for childhood environmental factors in non-cohabiting twins cannot be excluded. Some of the most concordant traits included height, telomere length, IL6 receptor levels, and platelet measures. One of the few traits that showed high correspondence in spouse pairs was cotinine level. This may not be surprising, since cotinine levels are reflective of smoking behavior. Concordance rates for smoking are generally high in spouses, both before and after marriage [40] and smoking is one of the cardiovascular risk factors with highest concordance among spouses [41].

The results demonstrate that substantial differences may exist in the relative contribution of genetic factors to the various components of a single biological system. The interaction between circulating IL6 and its soluble receptor is a case in point. IL6 is a potent pleiotropic cytokine secreted by T-cells and macrophages that regulates cell growth and differentiation and plays an important role in immune response. Interleukin 6 receptor (IL6R) forms a ligand–receptor complex with IL-6 that is capable of stimulating a variety of cellular responses including proliferation, differentiation and activation of inflammatory processes [42,43]. Raggi et al previously showed similar high heritability for IL6R [44], while other studies reported low to moderate heritability for IL6. IL6R level seems to be a stable individual characteristic with a genetic component [45], while IL6 level may be more responsive to environmental conditions.

A few extensions and limitations of our approach should be mentioned. The current paper provides a proof of principle in the proposed methodology combined with the unique study design, but includes only a snapshot of the variables available. However, the number of phenotypic traits that are examined here can be extended in number as well as towards categorical variables. The development of integrated phenotypic databases and increased use of electronic medical record systems will allow for richer and more comprehensive analyses to be carried out. In addition, longitudinal data can be incorporated. Following phenotypic markers in twins from birth, through adolescence, adulthood and old age and studying the change in their phenotypic landscape though time will be a very valuable extension of this work. Furthermore, validating some of the relationships and correlations presented here in a larger independent cohort would strengthen the findings. The current study compared MZ twin pairs, who share their genes and childhood environment, with spouse pairs, who share their adult environment. The impact of the shared environment in spouse pairs may differ depending on the time the spouses have been together and the extent to which they take part in similar activities. It would be interesting to investigate the similarity of the phenotypic profile as function of shared interests and cohabitation duration in a larger sample. Similarly we assume that sharing a household in adulthood implies that shared environment can affect phenotype. The effect of the shared as well as non-shared environment of the individuals at a young age or in utero is not captured by our study and is a limitation of the approach. Because there is such limited evidence from studies using the classical twin design (comparing MZ to DZ resemblance) that early common environment has an influence on the traits we study, we explored if the current shared environment has an influence. Both approaches seem to lead to similar conclusions: a main effect of shared environment is very hard to detect.

In conclusion, we present here a comprehensive analysis of the phenotypic profile and relationships between anthropometric, hematological, metabolic, and inflammatory traits of interest in twin pairs and spouse pairs. We show complex relationships between many of the phenotypic variables, forming metabolic and inflammatory clusters. The increased twin similarity demonstrates the importance of genetic factors while the larger concordance in spouse pairs compared to unrelated pairs and the comparison of cohabiting and non-cohabiting twins indicates that specific household factors may also play a role, albeit small, in the metabolic and immunological profile.

## Supporting Information

S1 Fig The distribution of phenotypic measurements (shown as sex-and age corrected residuals) in the total sample (N = 51). (PDF)

S2 Fig How closely do twins cluster together in comparison to unrelated individuals? For each twin pair the rank of how similar the other twin’s profile is in comparison to all the other phenotypic profiles in the cohort is displayed. Panel A shows the results on the actual data, panel B shows the results on a representative permutation of the data. For the majority of the twins, their twin pair falls within the top five most similar individuals based on their phenotypic profile.
(PDF)

S1 Table Partial Pairwise Correlations between Traits. This table contains all the significant partial pairwise correlation values between the measured phenotypic traits. The non-significant values were set to 0.
(XLSX)

S2 Table Pair-wise correlations and confidence intervals for each trait in each of the four comparison groups (MZ twin pairs living together, MZ twin pairs living apart, twin-spouse pairs, unrelated individuals) ordered by concordance in twins living together. (XLSX)
