# Supplementary figures and images for: Effect of Genome and Environment on Metabolic and Inflammatory Profiles
Source: PLoS One. 2015 Apr 8;10(4):e0120898. doi: 10.1371/journal.pone.0120898 (PMC4390246; doi:10.1371/journal.pone.0120898)

Sex and Age Corrected Data

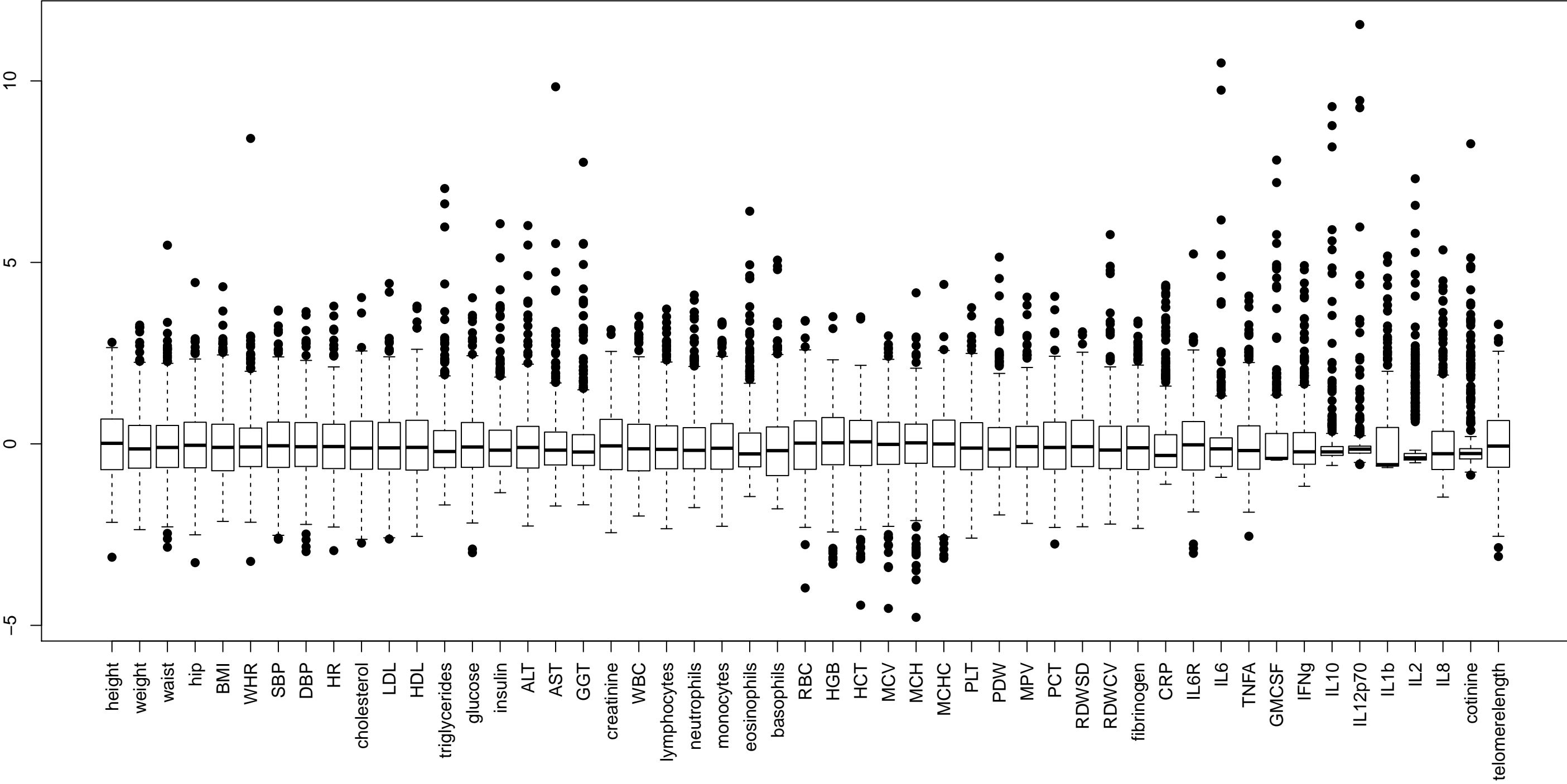

Supplement: S1 Fig — (PDF) [file pone.0120898.s001.pdf]

**A****Twin-Twin**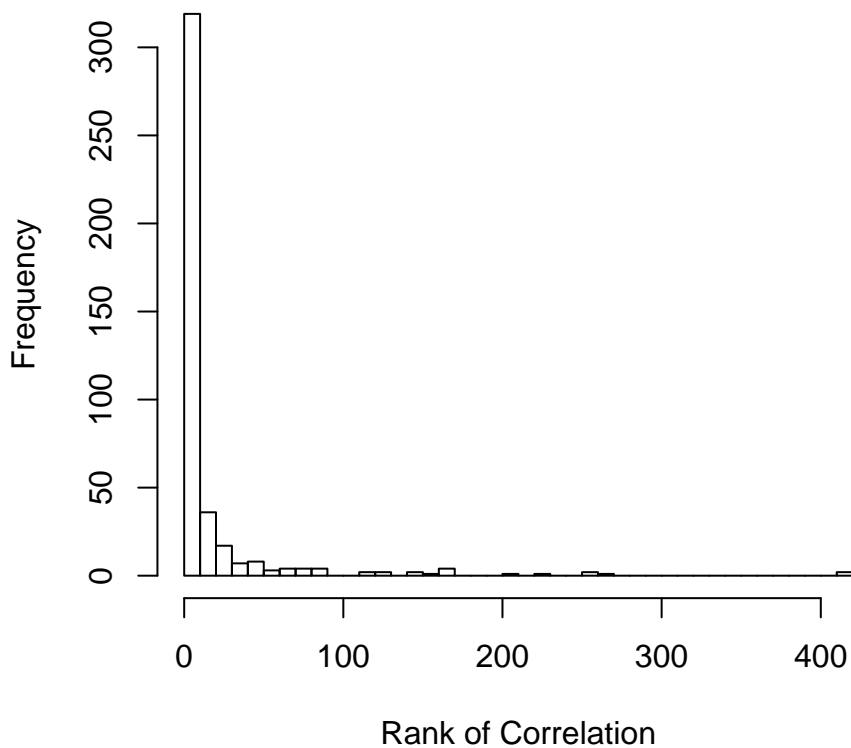**B****Randomized**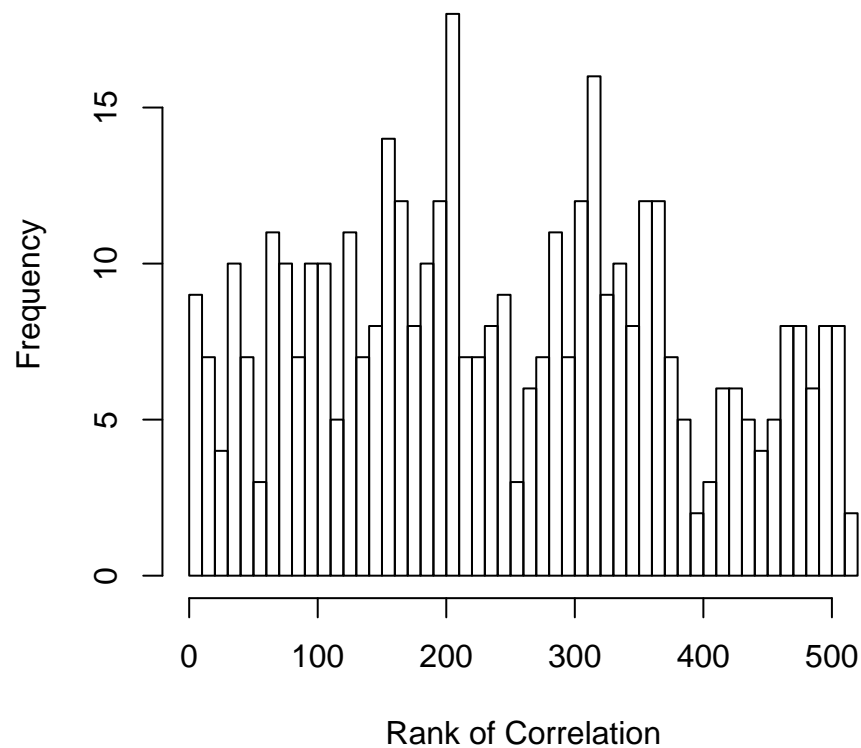

Supplement: S2 Fig — For each twin pair the rank of how similar the other twin’s profile is in comparison to all the other phenotypic profiles in the cohort is displayed. Panel A shows the results on the actual data, panel B shows the results on a representative permutation of the data. For the majority of the twins, their twin pair falls within the top five most similar individuals based on their phenotypic profile. (PDF) [file pone.0120898.s002.pdf]
